# Development of a Biopolymer-Based Anti-Fog Coating with Sealing Properties for Applications in the Food Packaging Sector

**DOI:** 10.3390/polym16121745

**Published:** 2024-06-20

**Authors:** Masoud Ghaani, Maral Soltanzadeh, Daniele Carullo, Stefano Farris

**Affiliations:** 1 Department of Civil, Structural & Environmental Engineering, School of Engineering, Trinity College Dublin, College Green, 2 Dublin, Ireland; 2 Food Packaging Laboratory, Department of Food, Environmental and Nutritional Sciences—DeFENS, University of Milan, Via Celoria 2, 20133 Milan, Italy; maral.soltanzadeh@unimi.it (M.S.); daniele.carullo@unimi.it (D.C.); stefano.farris@unimi.it (S.F.)

**Keywords:** biopolymers, anti-fog coating, pullulan, poly (acrylic acid), seal strength, sustainable packaging

## Abstract

The quest for sustainable and functional food packaging materials has led researchers to explore biopolymers such as pullulan, which has emerged as a notable candidate for its excellent film-forming and anti-fogging properties. This study introduces an innovative anti-fog coating by combining pullulan with poly (acrylic acid sodium salt) to enhance the display of packaged food in high humidity environments without impairing the sealing performance of the packaging material—two critical factors in preserving food quality and consumers’ acceptance. The research focused on varying the ratios of pullulan to poly (acrylic acid sodium salt) and investigating the performance of this formulation as an anti-fog coating on bioriented polypropylene (BOPP). Contact angle analysis showed a significant improvement in BOPP wettability after coating deposition, with water contact angle values ranging from ~60° to ~17° for formulations consisting only of poly (acrylic acid sodium salt) (P0) or pullulan (P100), respectively. Furthermore, seal strength evaluations demonstrated acceptable performance, with the optimal formulation (P50) achieving the highest sealing force (~2.7 N/2.5 cm) at higher temperatures (130 °C). These results highlight the exceptional potential of a pullulan-based coating as an alternative to conventional packaging materials, significantly enhancing anti-fogging performance.

## 1. Introduction

As a novel source of innovative materials, biopolymers have garnered significant interest over the past few decades, becoming a critical area of study in the materials science field [1]. In particular, the food packaging sector is rapidly evolving with the commercialization of these materials with enhanced and unprecedented functional properties [2]. Among others (e.g., barrier performance against gases and vapours, thermal stability, active attributes, optical properties, etc.), the anti-fog feature of packaging films has received considerable attention. This feature refers to the material’s ability to prevent the formation of tiny water droplets on the inner surface of packaging, a phenomenon that typically occurs due to environmental changes in temperature and humidity [3]. The newly formed droplets produce a foggy layer that alters the optical properties of the material, hindering the clear display of the food product by scattering light in various directions. This phenomenon occurs especially in fresh food and minimally processed vegetables during cold storage (i.e., refrigeration), whereby the transparency of the film significantly influences consumer choices [4,5].

Pullulan is a non-ionic exopolysaccharide (derived from *Aureobasidium pullulans*) with commendable characteristics, such as non-toxicity, biodegradability, and superior film-forming capabilities [6]. Pullulan’s distinctive structural attributes, such as the α(1−6) linkage between maltotriose units, yield peculiar characteristics of this biopolymer, such as high flexibility and solubility in water. Its molecular structure, adorned with hydroxyl groups, facilitates extensive intermolecular hydrogen bonding and super-hydrophilicity. These features were first harnessed to develop coatings with excellent oxygen barrier properties [7,8], even in high relative humidity environments [9]. Later on, one of the main drawbacks associated with pullulan, moisture sensitivity [10], has proved advantageous for the new generation of anti-fog coatings [11]. 

However, the effectiveness of anti-fog pullulan coatings for practical uses also depends on their ability to adequately seal coated material, as the coating is intended for the inner surface of the packaging film, typical in products such as fresh salads. While pullulan inherently possesses some sealing attributes, it does not rival the sealing strength of more conventional materials such as thermoplastic polymers (polyethylene or polypropylene). Therefore, the exploration and development of innovative pullulan-based films or coatings that concurrently offer anti-fogging efficacy and adequate sealing capabilities are needed.

This study introduces a ground-breaking anti-fog coating that combines the biopolymer pullulan with an acrylic component—specifically, poly (acrylic acid) sodium salt. This component was chosen for its negative charges (COO^−^) in an aqueous medium, which significantly enhances the sealing properties of the coating. Polyacrylic acid is characterised by sequences of negatively charged carboxylate groups (COO^−^). Similar to well-known ionomers, the possibility of ionic interactions culminates in its strength potential, including hydrogen bonding and chain entanglement [12].

Unlike previous studies that primarily focused on anti-fogging or sealing properties, this research integrates both functionalities into a single coating. This dual-functional coating offers a more compelling, functional alternative to existing market offerings, addressing critical concerns like fogging while preserving the visual appeal and integrity of the packaged food throughout its shelf life.

## 2. Materials and Methods

### 2.1. Materials

Pullulan powder (PF-20 grade, molecular weight ~200.000 Da), was procured from Hayashibara Biochemical Laboratories Inc., Okayama, Japan. Poly acrylic acid (PAA) sodium salt (molecular weight 2100 Da) was obtained from Sigma-Aldrich (Burlington, MA, USA). A primer solution containing 0.5 wt % aziridine (Michem^®^ Flex P2300) was acquired from Michelman International (Aubange, Belgium). The substrate for coating deposition, corona-treated bi-oriented polypropylene (BOPP) with a thickness of 20 ± 0.5 μm, was sourced from Taghleef Industries S.p.A. (S. Giorgio di Nogaro, Italy). The choice of this material accounts for its widespread use as a packaging material for fresh and ready-to-eat salads. Milli-Q water, exhibiting a resistivity of 18.3 MΩ cm, was employed throughout the experiment.

### 2.2. Film Preparation

Various concentrations of pullulan were added to PAA (22 wt% in water) solution under stirring conditions (600 rpm for 1 h) to achieve pullulan/PAA ratios of 1:3.78, 1:1.78, and 1:1.11. These formulations were designated as P25, P50, and P75, respectively, as reported in Table 1. Additionally, a solution of pure pullulan was labelled P100, and a solution solely consisting of PAA was identified as P0. Both P100 and P0 served as control specimens.

The surface of rectangular BOPP stripes (24 × 18 cm^2^) underwent corona treatment (Arcotec, Ülm, Germany) for surface activation to bolster the adhesion between the substrate and the coatings. To optimise this process, a primer layer consisting of 0.5 wt% aziridine homopolymer in a water/ethanol solution was uniformly applied to the BOPP surface after corona treatment using an automatic film applicator (model 1137, Sheen Instruments, Kingston, UK). This applicator was equipped with a steel rod and an engraved pattern, which facilitated the deposition of the primer layer with a wet thickness of 4.0 μm. The subsequent evaporation of the solvent was achieved by directing a steady, perpendicular flow of mild air (25.0 ± 0.3 °C for 2 min) at a distance of 40 cm from the applicator. Following this, a secondary layer comprising different pullulan/poly (acrylic acid sodium salt) solutions was deposited using a rod, ensuring a consistent wet layer thickness of 4.0 μm. For the initial four formulations (P0 to P75), a singular coating layer was applied. By contrast, for the P100 formulation (with a dry substance content of 11%), the coating process was repeated to attain a final layer thickness comparable to the P0 formulation, which had a dry substance content of 22%.

The application of both layers was conducted at a uniform speed of 2.5 mm s^−1^, following ASTM D823-18(2022)-Practice C [13]. The coated films were subsequently stored under controlled conditions (25.0 ± 0.3 °C in a desiccator) for a duration of 15 days before any analytical evaluations. This step ensured a controlled and gradual removal of residual moisture while avoiding any potential moisture uptake from the surrounding environment before the following tests. The coated films were uniform and clear, with no visible defects or irregularities. This uniform appearance indicates the successful application and adhesion of the pullulan/PAA coating on the BOPP substrate.

### 2.3. Thickness Determination

To ascertain the thickness of the layers coating the plastic films, a systematic approach was employed. Initially, a sample measuring 10 × 10 cm^2^ was sectioned, and its weight (*M*_1_) was accurately determined. Subsequently, the coating was removed from the base film using hot water at a temperature of 80 °C, and the weight of the stripped base film was recorded as *M*_2_. Adequate coating thickness was deduced using Equation (1):

(1) $$l=\frac{M_{1}-M_{2}}{\rho }\times 100$$

where, *M*_1_ represents the total mass per unit area of the plastic film inclusive of the coating (expressed in g/dm^2^); *M*_2_ signifies the mass per unit area of the plastic film alone (also in g/dm^2^); *ρ* denotes the density of the aqueous solution (measured in g/cm^3^). The thickness of the coating layer, *l*, in micrometers (μm), is determined by the known values of *M*_1_ − *M*_2_ [14].

### 2.4. Seal Strength Determination

Film strips measuring 2.5 cm × 10 cm^2^ were heat-sealed using a Polikrimper TX/08 thermal heat sealer (Alipack, Pontecurone, Italy), equipped with smooth plates. The temperature of the sealing plates varied from 80 °C to 130 °C in 10 °C increments. A microprocessor consistently regulated the preset temperature of each bar during the experiment. The sealing pressure was maintained at 4.5 bar with a dwell time of 1 s. The seal strength of the heat-sealed samples was quantitatively assessed via the T-peel test, conforming to the standardised protocol delineated in ASTM F88-07a [15]. This evaluation was conducted employing a dynamometer (model Z005, Zwick Roell, Ulm, Germany), equipped with a 5 kN load cell and interfaced with two clamps spaced 10 cm apart. The test was executed at a crosshead speed of 300 mm/min. Critical parameters, such as the peak load (indicating the maximum force required to break the joints) and strain energy (represented by the area under the load (N)—deformation (mm) curve up to the point of breakage), were accurately computed and extracted using TestXpert V10.11 Master software (Zwick Roell, Ulm, Germany), which provided a quantitative measure of seal strength.

### 2.5. Contact Angle Measurements

Contact angle analysis was conducted using an advanced optical contact angle apparatus (OCA 15 Plus, Data Physics Instruments GmbH, Filderstadt, Germany) outfitted with a high-resolution CCD camera and a high-performance digitizing adapter. SCA20 software (version 1.0) facilitated the contact angle measurements. Rectangular specimens, each measuring 5 × 2 cm^2^, were securely positioned and maintained in a flat orientation throughout the analysis using a specialised sample holder equipped with parallel clamping jaws.

The static contact angle of water in the air (θ, degrees) was meticulously determined by employing the sessile drop method. This process involved dispensing a droplet of Milli-Q water (18.3 MΩ cm) with a volume of 4.0 ± 0.5 μL onto the coated surface. The procedure followed was the so-called pick-up technique: A droplet, suspended from a needle, was gently positioned on the solid surface by elevating the sample stage until the solid/liquid interface was established. The measurements were conducted under controlled conditions at a temperature of 23 ± 1 °C and 50 ± 2% relative humidity (RH). To ensure uniform measurements, all droplets (ten per sample) were dispensed at a consistent height of 1 cm above the surface. The static contact angle was then recorded immediately following the droplet’s deposition (time zero, t0) and after a duration of three seconds (t3). This angle was identified as the one formed between the drop baseline and the tangent at the drop’s periphery.

### 2.6. Coefficient of Friction Measurements

The static and dynamic coefficient of friction (COF) values for the films were quantified in compliance with the ASTM D1894-14 standard using a dynamometer (model Z005, Zwick Roell, Ulm, Germany) equipped with a 100 N load cell [16]. The COF assessments, encompassing both coating-to-coating and coating-to-metal interactions, were conducted in a controlled laboratory environment, maintaining a stable temperature of 23 ± 2 °C and relative humidity (RH) of 50 ± 2%. These values represent the mean of three individual measurements to ensure accuracy and repeatability.

### 2.7. Optical Properties

Haze measurements were performed across a wavelength spectrum of 780–380 nm, adhering to the guidelines of ASTM D1003-00 [17]. These measurements were facilitated by a UV–Vis high-performance spectrophotometer (Lambda 650, PerkinElmer, Waltham, MA, USA) coupled with a 150 mm integrated sphere, allowing diffuse transmitted light to be captured. Haze is quantified as the percentage of light that, upon transmission, deviates by an angle of more than 2.5° from the incident beam’s direction. This parameter is of significant commercial relevance as it directly influences the contrast and visibility between objects viewed through the film. These values represent the mean of three individual measurements, ensuring statistical robustness and reliability of the data.

### 2.8. Statistical Analysis

Determining statistical significance among the mean values was carried out via one-way analysis of variance (ANOVA) using Statgraphics Plus 4.0 software. Where applicable, the mean values were compared using Student’s *t*-test with a predefined significance threshold set at *p* < 0.05. This methodological approach ensured a rigorous statistical evaluation of the data and discerned significant differences in mean values across the groups.

## 3. Results and Discussion

### 3.1. Thickness Analysis

**Figure 1:**
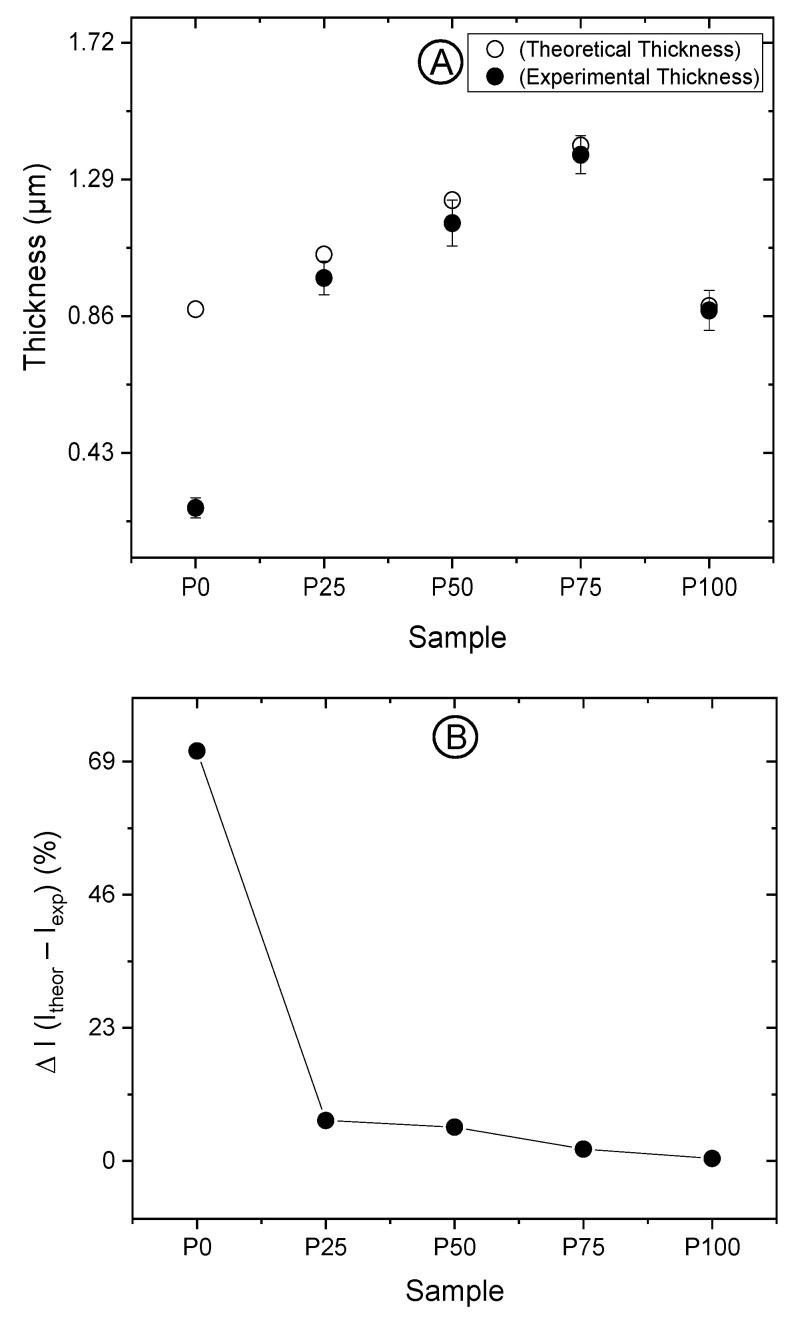
(**A**) Comparison between theoretical and experimental coating thicknesses for samples P0, P25, P50, P75, and P100. (**B**) Percentage variation between theoretical and experimental coating thicknesses for the different samples.

Accurate thickness analysis ensures uniformity and consistency in coatings, which are crucial for maintaining barrier performance, mechanical strength, and optical clarity. Moreover, precise thickness measurement is particularly important for determining haze, as variations can significantly impact the optical properties of the coatings. Table 2 outlines experimental thickness measurements derived using the methodology described in Section 2.3, exhibited deviations from the theoretical thickness (*TT*) values, and calculated based on the proportion:

4:100 = *TT*:*DW* (2)

where, 4 μm symbolises the thickness of the coating applied with a steel horizontal rod to achieve a wet coating thickness of approximately 4 μm, and *DW* represents the dry weight content of the solution deposited on the substrate. The comparative analysis between nominal (theoretical) and actual (experimental) thickness values for each sample is presented below:

According to the statistical analysis, significant differences in thickness were observed across various samples. However, no considerable variance was noted between the P25 and P50 formulations, and between the P25 and P100 formulations. Figure 1A graphically represents the divergence of actual thickness from theoretical values, indicating that experimentally obtained grammage values tend to align more closely with theoretical grammage values when the coatings have an increased pullulan concentration. This finding can be explained based on coating solutions’ rheological properties. Similarly, Figure 1B reveals that the percentage difference between theoretical and experimental thickness diminishes and eventually nullifies with an increasing pullulan/acrylic lacquer ratio.

The trend between nominal and actual coating layer thickness has two plausible explanations. On the one hand, by increasing the pullulan concentration, the coating solution can leave the engraved pattern of the deposition rod easier than formulations based on higher amounts of acrylic, whose charged nature explains higher interactions with the metallic rod, leading to a lower amount of coating solution deposited on the plastic substrate. For practical purposes, this aspect must be considered, as it would require a different engraved pattern (i.e., able to deposit an extra amount of coating solution) or increasing the solids content of the coating solution to achieve the target grammage. On the other hand, the negative charges of the acrylic component engage in electrostatic interactions with the positively charged primer (aziridine), fostering an attraction that makes coating removal from the substrate difficult; consequently, the coating thickness is underestimated (see Equation (1)). By contrast, pullulan, a molecule devoid of electric charge, forms weaker interactions with the substrate, easing its removal. This characteristic is particularly evident in the near-complete detachment of the pure pullulan coating (P100), where the experimental grammage values closely match the theoretical ones.

### 3.2. Sealing Analysis

**Figure 2:**
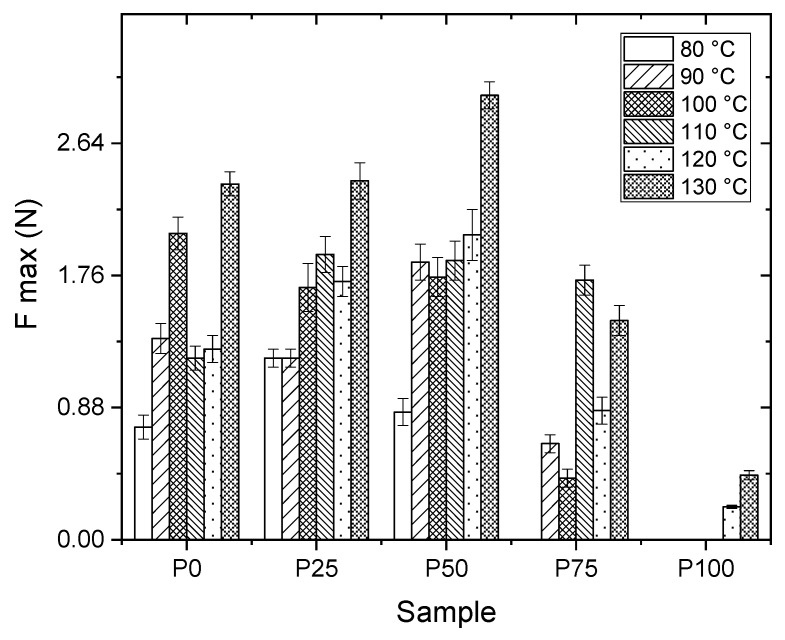
Seal strength comparison at various temperatures for samples P0, P25, P50, P75, and P100.

As illustrated in Figure 2, the P100 formulation exhibits a markedly lower maximum sealing force than other formulations. This observation underscores the fact that pullulan, in its pure form and devoid of additives, lacks inherent sealing properties—a characteristic that remains unchanged even under elevated temperatures. Despite these thermal conditions, the sealing performance of the P100 formulation cannot surpass even the least effective performance of the P0 formulation, highlighting the pivotal role of the acrylic component in conferring sealability.

Sealing is a macroscopic phenomenon where molecular dynamics play a crucial role. At the glass transition temperature (*T*_g_), there is a significant increase in molecules’ mobility, marking the transition from a glassy, rigid state to a more flexible state. This mobility enhancement, as temperatures escalate towards the melting temperature (*T*_m_), allows molecules to blend, diffuse and, upon cooling, solidify into a new position, facilitating the formation of a stable seal. The interactions between ionic charges help maintain this stability, especially in materials where ionic bonds contribute to the structure. While repulsive forces between like charges exist, their impact is often overshadowed by cohesive forces that drive the sealing process. This balance of forces ensures the material’s integrity and functionality at the macroscopic level [12,18].

Furthermore, Figure 2 delineates a general pattern where higher seal strength is achieved with coatings subjected to a sealing temperature of 130 °C, implying that sealing properties are enhanced at elevated temperatures. The same graph also indicates that optimal performance (~2.7 N/2.5 cm) is attributed to the P50 formulation, which aligns with the highest sealing force values. Notably, up to a temperature of 110 °C, no statistically significant differences (*p* < 0.05) are discernible between this formulation and the others, suggesting a nuanced interaction between temperature, formulation composition, and the resulting sealing efficacy. The enhanced seal strength indicates adequate adhesion between the coating and the BOPP substrate mediated by the presence of aziridine homopolymer. Additionally, uncoated BOPP seal strength was measured to be ~5.5 N/2.5 cm, providing a reference for comparison. This finding indicates that pullulan/PAA coatings still demonstrate adequate sealing performance despite a lower seal strength than uncoated BOPP.

### 3.3. Contact Angle Analysis

The data presented in Table 3 elucidate specific trends and relationships between contact angle measurements at the initial time (t = 0 s) and after three seconds (t = 3 s). The deposition of the coating yielded a decrease in the water contact angle of the plastic substrate (BOPP), which is approximately 80° [19]. The significant reduction in water contact angles after coating deposition indicates enhanced wettability and suggests an even distribution of the coating on the BOPP substrate.

An inverse correlation between the pullulan concentration in the coating and the contact angle is evident, notably at t = 3 s, as proven by statistically significant differences (*p* < 0.05) among the various formulations. Additionally, the contact angle at t = 0 s invariably exceeded t = 3 s. The correlation mentioned above can be rationalised by investigating the chemical composition of the coating constituents, which can shed light on why pullulan and polyacrylic acid behave differently when in contact with water.

Pullulan, characterised by an abundance of hydroxyl (OH) groups along its chain, exhibits pronounced hydrophilicity. These OH groups engage in hydrogen bonding with water droplets on the coating, promoting uniform distribution and resulting in reduced contact angle values. Conversely, polyacrylic acid, consisting primarily of a non-polar hydrocarbon chain, exhibits a predominantly hydrophobic nature, except for its polar carboxylate groups. This configuration results in water droplets minimizing their surface area when in contact with a prevalently hydrophobic coating. They adopt a more spherical geometry and, consequently, higher contact angle values.

**Figure 3:**
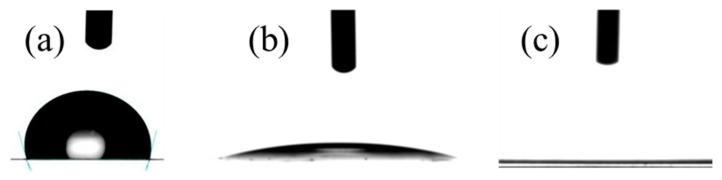
Water contact angle of (**a**) polypropylene film neutral (no surface treatment); (**b**) anti-fog coating (formulation P100) immediately after depositing the water droplet; (**c**) anti-fog coating (formulation P100) after ~10 s of depositing the water droplet.

Figure 3 presents a detailed visualization of how a droplet’s geometry evolves over time. This evolution, marked by a gradual reduction in the contact angle, is primarily influenced by two fundamental interactions at the interface of the solid surface and liquid: absorption and spreading [11]. The morphology of the droplet may theoretically be influenced by additional factors, including the evaporation of the liquid from the droplet and the physical expansion of the substrate due to moisture absorption. However, their effects were deemed negligible in this specific analysis due to a lack of experimental confirmation under the conditions being studied.

The absorption process is intricately linked to the physical structure of the substrate, notably the presence of microscopic channels or capillaries that enable liquid droplets to permeate the substrate. This infiltration leads to a notable decrease in the droplet’s overall volume and the area of its base, which is in direct contact with the substrate. Spreading, however, describes the dynamic process by which the droplet extends outward across the substrate surface. This spreading is significantly influenced by the physical characteristics of the substrate, such as its smoothness and chemical affinity for water, resulting in the droplet adopting a more flattened shape. This change increases the contact area of the droplet’s base with the substrate but does not affect its volume. Notably, on pullulan-based substrates, a biopolymer known for its high hydrophilicity, spreading occurs at a markedly faster rate than absorption [11]. Given the negligible role of absorption in this context, spreading emerges as the sole mechanism driving change at the contact angle, underscored by pullulan’s exceptional water-attracting capabilities and the substrate surface’s specific morphological features designed to facilitate this interaction.

### 3.4. Coefficient of Friction (COF)

The COF analysis has practical relevance for assessing a material’s suitability to run smoothly on packaging equipment during converting and packaging operations. Too high COF values can determine the blocking effects of the reels, eventually leading to unwanted ruptures of the webs and severely impacting the overall throughput of the process.

The COF results obtained in this work revealed insightful patterns in the interaction between coatings and metal surfaces. The COF values for coating/metal interfaces were higher in formulations containing the acrylic component than in the P100 formulation, which solely consists of pullulan. According to the statistical evaluation presented in Table 4, COF values on metal substrates did not exhibit statistically significant differences among acrylic-containing formulations, except for dynamic coating/metal COF measurements. Notably, the P100 formulation demonstrated significantly lower static and dynamic COF values than its counterparts.

The presence of the acrylic component markedly influenced COF values, which can be attributed to the interaction between negative charges of the polyacrylic acid, which somehow promote interactions with the metallic surface. These interactions restrict the movement of the sled covered with the coated film that slides on the metal surface. By contrast, pullulan, being an uncharged molecule, does not form such interactions with the metal surface, resulting in lower COF values.

A contrasting trend is observed when comparing coating/metal and coating/coating COF values. The P100 formulation exhibits significantly higher COF values in coating/coating interactions than acrylic-containing formulations. This difference is due to the unique characteristics of acrylic-based formulations, where the presence of negatively charged particles creates a repelling effect, leading to lower friction between the coating layers. This repelling action allows acrylic formulations to have lower COF values. On the other hand, the P100 formulation, comprised solely of pullulan, lacks such repelling forces and has a smoother surface. This lack of repulsion, combined with a smoother surface, increases resistance when coatings touch each other, raising COF values. This contrast highlights the significant impact of acrylic’s negative charge and the coatings’ physical qualities on the friction observed in coating/coating interactions.

### 3.5. Optical Properties

**Figure 4:**
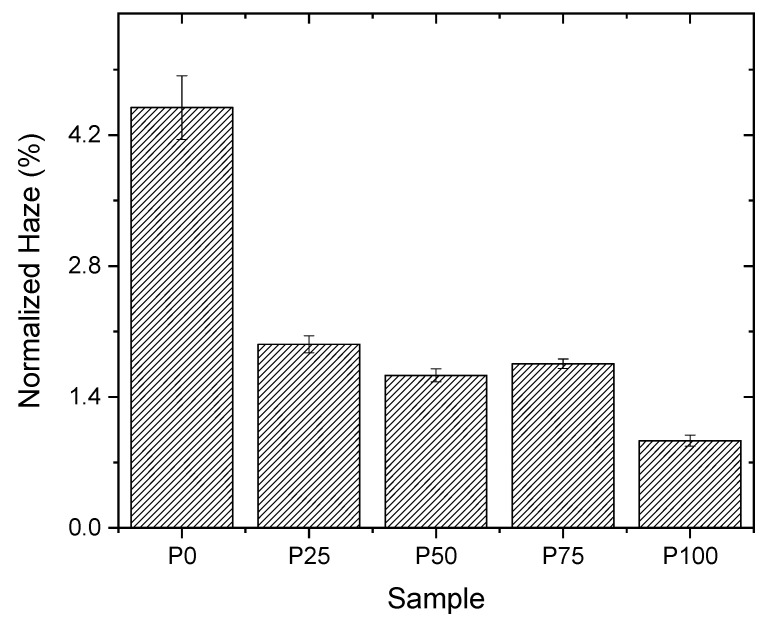
Normalised haze percentages at 1 µm coating thickness for samples P0, P25, P50, P75, and P100.

The data presented in Table 5 indicate no notable statistical differences in the haze values among the different formulations, except for the P75 and P100 formulations. Additionally, all measured values are below the 3% threshold, a value widely recognised for plastic materials in food packaging to ensure sufficient transparency for visual inspection of the contents [20]. The normalised haze values presented in Figure 4 correspond to an assumed coating thickness of 1 μm based on the premise that haze variation is directly proportional to thickness. The calculation of opacity values for a 1 μm thickness was executed using the following proportions:

Haze_s_:Thickness_s_ = Haze_n_:1 μm (3)

Here, *Haze*_s_ signifies the experimentally determined opacity value (%), *Thickness*_s_ denotes the coating’s thickness as measured by the weight method (μm), and *Haze*_n_ represents the normalised opacity value for a 1 μm thick coating (%). Consequently, the formula to deduce the normalised haze is articulated as follows:

(4) $${Haze}_{n}=\frac{\left({Haze}_{s}\times 1\right)}{{Thickness}_{s}}$$

Figure 4 reveals a direct correlation between the increase in haze values and the augmented acrylic content in the coating. This trend is potentially attributable to the emergence of scattering centres within the polyacrylic acid formed by the clustering of acrylic molecules, which scatter the incident light and increase opacity. The P75 formulation exhibits peculiar behaviour due to its composition, featuring less acrylic content but higher pullulan content, rendering it more resistant to dissolution. Therefore, it is reasonable to infer that the formation of partially solubilised pullulan aggregates, observable in optical analyses, contributes to heightened opacity and serves as scattering centres. For reference, the haze of uncoated OPP is measured at 0.71 ± 0.05%. This baseline measurement demonstrates that while the addition of pullulan/PAA coatings increase haze, coatings still maintain transparency levels acceptable for food packaging applications.

## 4. Conclusions

This study introduces a pioneering approach to solving the problem of fog formation on food packaging films by combining pullulan, a naturally derived biopolymer, with poly (acrylic acid sodium salt) to forge a novel anti-fog coating. The research demonstrated that this unique combination not only mitigates the fogging phenomenon by leveraging pullulan’s inherent hydrophilicity but also elevates the seal strength critical for maintaining food freshness, as evidenced by the enhanced sealing properties observed at varying temperatures. Notably, the investigation highlighted the formulation’s superior anti-fogging performance, which was quantitatively affirmed through reduced contact angles, indicating more efficient moisture management on the packaging surface. Additionally, including poly (acrylic acid sodium salt) introduced negative charges that improved the coating’s adhesion and sealing capabilities, a notable advancement over conventional materials. Optical properties analysis further validated the formulation’s efficacy and maintained transparency within acceptable limits, thus ensuring product visibility without compromising sustainability. This research not only advances sustainable biopolymer-based packaging but also highlights the importance of innovation in solving the food industry’s challenges, setting a foundation for future advancements in eco-friendly and functional packaging solutions. Future work will include comprehensive anti-fogging tests under simulated conditions and on real food products to thoroughly validate the coating’s effectiveness. The effectiveness and clarity of this research will enhance its commercial applicability to food packaging.

## Figures and Tables

**Table 1:** Composition and dry matter content of pullulan/PAA coating solutions.

Code	Pullulan (g)	PAA Solution (g)	Dry Matter of the Coating Solution (%)	Pullulan/PAA
P0	0	10	22	0:1
P25	0.55	9.45	26.29	1:3.78
P50	1.1	8.9	30.58	1:1.78
P75	1.65	8.35	34.87	1:1.113
P100	1.1	0	11	1:0

**Table 2:** Nominal and actual coating thicknesses for samples P0, P25, P50, P75, and P100.

Sample	Nominal Thickness (μm)	Actual Thickness (μm)
P0	0.88	0.26 ^a^ ± 0.03
P25	1.05	0.98 ^bc^ ± 0.05
P50	1.22	1.15 ^c^ ± 0.07
P75	1.39	1.37 ^d^ ± 0.06
P100	0.88	0.88 ^b^ ± 0.06

Data for actual thickness are the mean of triplicate measurements ± SD values. Superscripts in this column indicate the results of a statistical comparison using a post hoc test. Samples with the same or overlapping superscript letters are not significantly different (*p* > 0.05).

**Table 3:** Contact angle measurements at the initial time (t = 0 s) and after three seconds (t = 3 s) for various pullulan concentrations in the coating formulations.

Sample	Contact Angle (^°^)	
	t = 0 s	t = 3 s
P0	61.19 ^a^ ± 1.79	58.83 ^d^ ± 1.43
P25	59.00 ^a^ ± 1.89	55.17 ^e^ ± 2.13
P50	56.35 ^b^ ± 1.05	53.30 ^f^ ± 0.95
P75	60.87 ^a^ ± 1.04	37.62 ^g^ ± 1.89
P100	44.25 ^c^ ± 5.68	16.81 ^h^ ± 1.24

Data are the mean of triplicate measurements ± SD. Different letters as superscripts in each column represent significant differences between means (*p* < 0.05) using a post hoc test.

**Table 4:** Coefficient of friction (COF) measurements for coating/metal and coating/coating interfaces at static and dynamic states across various formulations.

Sample	COF (Coating/Metal)		COF (Coating/Coating)	
	Static	Dynamic	Static	Dynamic
P0	0.45 ^a^ ± 0.03	0.31 ^cd^ ± 0.04	0.71 ^e^ ± 0.02	0.61 ^g^ ± 0.03
P25	0.45 ^a^ ± 0.01	0.33 ^d^ ± 0.02	0.68 ^e^ ± 0.02	0.61 ^g^ ± 0.03
P50	0.46 ^a^ ± 0.02	0.32 ^cd^ ± 0.02	0.72 ^e^ ± 0.06	0.62 ^g^ ± 0.01
P75	0.45 ^a^ ± 0.02	0.35 ^d^ ± 0.01	0.77 ^e^ ± 0.03	0.62 ^g^ ± 0.02
P100	0.39 ^b^ ± 0.01	0.28 ^c^ ± 0.01	1.59 ^f^ ± 0.68	0.72 ^h^ ± 0.04

Data are the mean of triplicate measurements ± SD. Different letters as superscripts in each column represent significant differences between means (*p* < 0.05) using a post hoc test.

**Table 5:** Haze percentage measurements for samples P0, P25, P50, P75, and P100.

Sample	Haze (%)
P0	1.15 ^ab^ ± 0.14
P25	1.92 ^ab^ ± 0.96
P50	1.88 ^ab^ ± 0.09
P75	2.40 ^b^ ± 1.56
P100	0.82 ^a^ ± 0.12

Data are mean of triplicate measurements ± SD. Different letters as superscripts in each column represent significant differences between means (*p* < 0.05) using a post hoc test.

## Data Availability

Data are contained within the article.
